# A Comparison of the Crystal Structures of Eukaryotic and Bacterial SSU Ribosomal RNAs Reveals Common Structural Features in the Hypervariable Regions

**DOI:** 10.1371/journal.pone.0038203

**Published:** 2012-05-31

**Authors:** Jung C. Lee, Robin R. Gutell

**Affiliations:** Center for Computational Biology and Bioinformatics, Institute for Cellular and Molecular Biology, and Section of Integrative Biology, The University of Texas at Austin, Austin, Texas, United States of America; Cairo University, Egypt

## Abstract

While the majority of the ribosomal RNA structure is conserved in the three major domains of life – archaea, bacteria, and eukaryotes, specific regions of the rRNA structure are unique to at least one of these three primary forms of life. In particular, the comparative secondary structure for the eukaryotic SSU rRNA contains several regions that are different from the analogous regions in the bacteria. Our detailed analysis of two recently determined eukaryotic 40S ribosomal crystal structures, *Tetrahymena thermophila* and *Saccharomyces cerevisiae*, and the comparison of these results with the bacterial *Thermus thermophilus* 30S ribosomal crystal structure: (1) revealed that the vast majority of the comparative structure model for the eukaryotic SSU rRNA is substantiated, including the secondary structure that is similar to both bacteria and archaea as well as specific for the eukaryotes, (2) resolved the secondary structure for regions of the eukaryotic SSU rRNA that were not determined with comparative methods, (3) identified eukaryotic helices that are equivalent to the bacterial helices in several of the hypervariable regions, (4) revealed that, while the coaxially stacked compound helix in the 540 region in the central domain maintains the constant length of 10 base pairs, its two constituent helices contain 5+5 bp rather than the 6+4 bp predicted with comparative analysis of archaeal and eukaryotic SSU rRNAs.

## Introduction

While the ribosome is the site for protein synthesis, a function that is essential for all organisms spanning the entire tree of life, it is the ribosomal RNA (rRNA) that is directly associated with peptidyl transferase and decoding [1], [2], [3]. A comparison of the SSU rRNA sequences and their comparative structures reveals that a significant portion of their structure is conserved in all known organisms. Other regions are conserved within each of the three primary phylogenetic domains –archaea, bacteria, and eukaryotes, but differ between them [4], [5]. In comparision with the archaea and bacteria, several of the nine major variable regions (V1–V9) in the SSU rRNA [6] have large insertions in organisms within the eukaryotic phylogenetic domain [7]. While it is properly assumed that the SSU rRNA secondary structure that is conserved in eukaryotes, archaea, and bacteria will form the same three-dimensional structure, a more challenging question is if any structural elements in the eukaryotic variable regions with no obvious similarility in secondary structure in the bacteria can form a similar three-dimensional structure.

Comparative analysis has been used to accurately predict the secondary structure and a few tertiary structure interactions in numerous RNAs [8], including the bacterial *Thermus thermophiles* SSU rRNA [9]and an archaeal *Haloarcula marismortui* LSU rRNA [10]. To assess the accuracy of our comparative structure models for the eukaryotic SSU rRNAs [7], two recent near-atomic-resolution crystal structures of the eukaryotic ribosomal subunits, *Tetrahymena thermophila* 40S [11] and *Saccharomyces cerevisiae* 80S [12], were analyzed to determine not only their rRNA secondary and tertiary structure interactions, but also the ribosomal protein binding sites onto the rRNA. The results from this analysis were the foundation for the detailed comparison between the two eukaryotic 40S structures and the bacterial 30S structure from *Thermus thermophilus* [9].

## Results

### Accuracy of the eukaryotic *Tetrahymena thermophila* and *Saccharomyces cerevisiase* SSU rRNA comparative structures and a general comparison between the eukaryotic and bacterial SSU rRNA crystal structures

**Figure 1:**
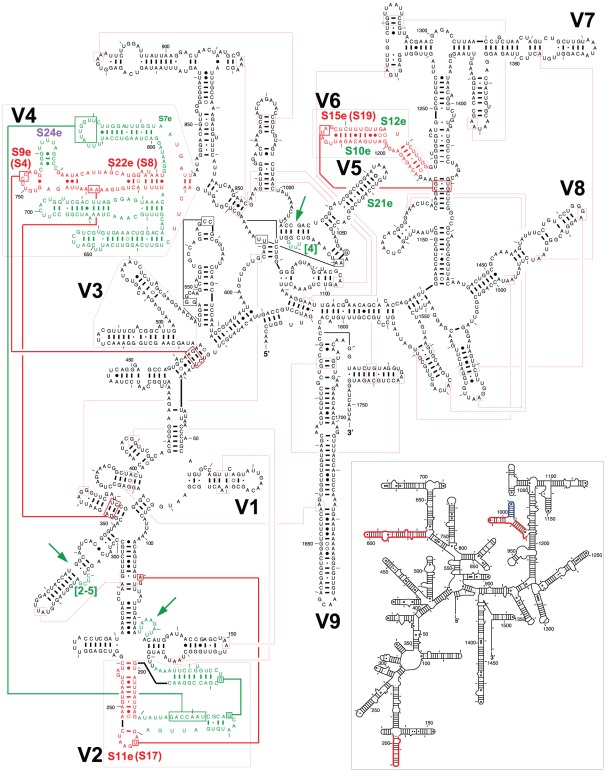
Comparison of the eukaryotic and bacterial SSU rRNA secondary structures. The base pairings, helices other RNA structural elements, and relevant ribosomal proteins in the *T. thermophila* 40S crystal structure are mapped onto the *T. thermophila* SSU rRNA comparative secondary structure diagram [7]. The nucleotides in the eukaryotic-specific structural elements are colored green, while the nucleotides colored red are structural elements in the variable regions that are analogous to helices in the bacterial structure. The V2, V4, and V6 variable regions are colored red while the bacterial-specific helix in V6 is shown in blue on the bacterial *Thermus thermophilus* SSU rRNA (inset). The long-range tertiary contacts maintained in both the eukaryotic and the bacterial SSU rRNA are shown with red lines, while those specific for the eukaryotic 18S rRNA in green lines; the tertiary contacts specifically associated with V2, V4, and V6 are shown with thicker lines. The ribosomal proteins common between eukaryotes and bacteria are shown in red, with their bacterial equivalents in parentheses, while those present only in eukaryotes in green and those present in archaea and eukaryotes in purple. The sequence insertions in the eukaryotic SSU rRNAs are highlighted in green with green arrows and numbers indicating the number of inserted nucleotides.

Our analysis revealed that the SSU rRNA structure in the 40S ribosomal subunit from *Tetrahymena thermophila* [11] is nearly identical to that in the 40S ribosomal subunit from *Saccharomyces cerevisiae* [12]. Analogous to the previous comparison between the comparative and crystal structures for the *Thermus thermophilus* SSU rRNA and the *Haloarcula marismortui* LSU rRNA [8], nearly all of the base pairs in both *T. thermophila* and *S. cerevisiae* comparative secondary structure models are present in their respective crystal structures as summarized in **Figure 1** for the *T. thermophila* SSU rRNA structure. This includes the part of the SSU rRNA structure that is conserved in the archaea, bacteria, and eukaryotes, and the proposed base pairs in the *T. thermophila* that are unique to the eukaryotes. The major structural elements generally characteristic of the eukaryotes are highlighted in red and green in **Figure 1**. These are (numbering refers to *E. coli* SSU rRNA):

1. Many non-canonical base pairs in the 39–46/395–403 helix.
2. An insertion of at least a few nucleotides at position 143.
3. The replacement of a single helix between positions 179 and 198 with two helices. This V2 region and its interaction with the V4 region is discussed below.
4. An insertion of 2–5 nucleotides between positions 249 and 252.
5. The V3 region, between positions 404–499 has two helices. While both are different from the analogous helices in bacteria and archaea, the second helix, closer to the 500–545 compound helix, is very irregular with several non-canonical base pairs and bulge nucleotides.
6. The V4 region is between positions 588 and 652. This V4 region is discussed below.
7. An insertion of four nucleotides between positions 876 and 877.
8. The V6 region is between positions 991 and 1046. Three helices occur in the bacteria while two are present in the archaea and the eukaryotes. This V6 region is discussed below.

All of the tertiary structure interactions in both eukaryotic SSU rRNA crystal structures that are not associated with eukaryotic specific structural elements are also present in the bacterial SSU rRNA crystal structure (**see Figure S1**). However, not all of the tertiary structure interactions were discerned in the two eukaryotic SSU rRNA crystal structures. As discussed below, a few of the tertiary structure interactions with the eukaryotic specific regions of the SSU rRNA were part of the rationale for our determination that specific structural elements in the two eukaryotic SSU rRNA structures were equivalent to helices in the bacterial SSU rRNA (see next section).

### Determination of the secondary structure in the V4 region that was unstructured in the comparative model and the identification of the eukaryotic equivalent structural elements for the bacterial helices in V2, V4, and V6

The V2 region (between nucleotide positions 179 and 197, *Escherichia coli* numbering) forms one helix in both bacteria and archaea. The length of this helix usually correlates with the presence or absence of an extra nucleotide at position 130 [13]. A single bulge nucleotide at position 130 is associated with a three base pair version of this helix while two bulge nucleotides at this position is associated with a 10 base pair helix. A tertiary base-base interaction was identified between the first nucleotide in the two nucleotide bulge loop and the first nucleotide of the CUUG tetraloop capping the 10 base pair helix in the bacterial *T. thermophilus* crystal structure [9].

**Figure 2:**
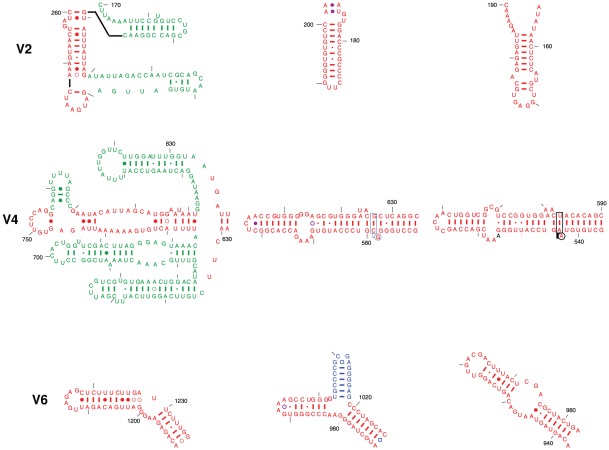
Gallery for the three hypervariable regions V2, V4, and V6 from the eukaryotic *T. thermophila* 18S, bacterial *T. thermophilus* 16S, and archaeal *Haloarcula marismortui* 16S rRNAs. The coloring scheme is the same as in Fig. 1. The helices shared by the bacterial 16S and eukaryotic 18S rRNAs are shown in red, while the eukaryotic-specific and bacterial-specific helices are shown in green and in blue, respectively.

The majority of the eukaryotes in the crown region of the phylogenetic tree including *Saccharomyces* and *Tetrahymena* contain approximately 90 nucleotides that form two compound helices in the V2 region in the comparative structure models [7]. These helices are present in the two eukaryotic crystal structures as noted previously [11]. The segments of the second compound helix (colored red in **Figures 1** and **2**) are equivalent to the bacterial and archaeal helix, based on: (1) the analogous tertiary structure interaction described earlier between position 130 and the hairpin loop and (2) the lonepair helical tip of the equivalent eukarytotic structure in V2 interacts with S11e as its bacterial counterpart interacts with S17 (**Figure 1**). The hairpin loop is formed from a lone base pair on the 3′ side of the internal loop of this compound helix.

The V4 region (nucleotide positions 588–652, *Escherichia coli* numbering) that forms a single compound helix in the bacterial and archaeal SSU rRNA with approximately 55 nucleotides is replaced in many of the eukaryotes in the crown region of the phylogenetic tree, with approximately 220 nucleotides. The first 100 or so nucleotides form two compound helices while approximately 120 consecutive nucleotides are unpaired in the Gutell lab's *Saccharomyces cerevisiae* and *Tetrahymena thermophila* comparative SSU rRNA secondary structure models [7]. Secondary structure models for this region of eukaryotic SSU rRNAs have been proposed by several groups [14], [15], [16], [17], [18], [19], [20], [21], [22]. De Wachter's pseudoknot structure [6] has been accepted by many as the correct secondary structure. No helical structure in these secondary structure models are identical to the compound helix in the 588–652 region of the bacterial and archael SSU rRNA. The 18S crystal structure of the *T. thermophila* 40S ribosomal subunit recently indicated the formation of four helical elements in this region [11].

The first two helices in the comparative structure model in the V4 region are present in the 40S ribosomal subunits for the two eukaryotic crystal structures. The last helix in the eukaryotic V4 region was not proposed in the Gutell lab's comparative structure but was proposed in some of the comparative structure models from other labs [14], [16], [17], [18], [19], [20], [21], [23]. The 5′ segment of the unstructured nucleotides in the Gutell lab's eukaryotic SSU rRNA comparative structure model contains numerous non-canonical base pairs to form several irregular helices in the eukaryotic crystal structure (**Figures 1** and **2**). That segment (colored red in **Figures 1** **and** **2**) are equivalent to the bacterial and archaeal compound helix between positions 588–652. This association is based on: (1) two analogous tertiary structure interactions, the first between the internal loop and base pairs 291:309 and 292:308 and the second between the hairpin loop closed by a single non-canonical base pair and the base pairs 41:401 and 42:400 (**Figure 1**), and (2) the interactions between the hairpin loop closed by a single non-canonical base pair and the stem of the equivalent eukaryotic helix with S9e and S22e, respectively, as their bacterial counterparts interact with S4 and S8, respectively (**Figure 1**).

A eukaryotic-specific long-range pseudoknot helix was proposed between the V2 and V4 hypervariable regions of eukaryotic SSU rRNA [24], based on the *S. cerevisiae* 80S cryo-EM structure and comparative analysis of the eukaryotic SSU rRNA sequences [25]. Subsequent experimental analysis supports this prediction [26]. This long-range pseudoknot helix connecting the V2 and V4 regions is at the lower back of both eukaryotic ribosomal small subunit crystal structures.

The V6 region is between positions 991 and 1046 in the SSU rRNA. As noted earlier, three helices are in bacteria, and two in the archaea and eukaryotes (**Figure 2**). The two helices in the *Tetrahymena* and *Saccharomyces* SSU rRNA comparative structure models were observed in the two eukcaryotic *T. thermophila* and *S. cerevisiae* ribosomal crystal structures. While the first helix 996–1003/1037–1045, is analogous in the three phylogenetic domains, the bacterial 1006–1012/1017–1023 helix is equivalent to the second helix in the eukaryotes (colored in **Figures 1** and **2**). This association is based on: (1) one analogous set of tertiary structure interactions between the hairpin loop of the 1006–1012/1017–1023 like helix and the base pairs 986:1219 and 987:1218 (**Figure 1**) and (2) the interactions between this helix and the eukaryotic ribosomal protein S15e, which is equivalent to the bacterial protein S19 (**Figure 1**).

### Role of eukaryotic-specific ribosomal proteins in eukaryotic SSU rRNA

Comparative analysis of the ribosomal proteins identified four ribosomal proteins that are only present in all eukaryotic organisms - S7e, S10e, S12e, and S21e [27]. S7e interacts with a part of V4, S10e and S12e interact with a U-turn in V6, and S21e interacts with V5 (**Figure 1**). Of particular interest is S12e which is located at the same spatial position occupied by the bacterial-specific helix in the V6 region, suggesting that this segment of RNA is replaced by a protein during the evolution of the ribosome structure.

### Maintenance of helix length in the coaxially stacked helices in the 540 region

**Figure 3:**
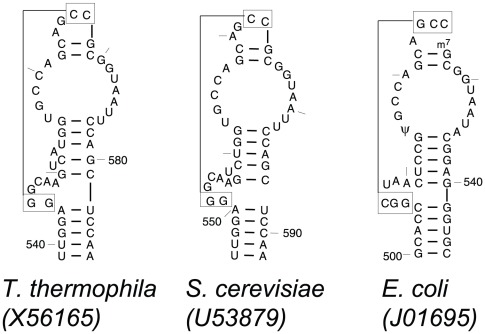
Comparison of the 540 regions in the eukaryotic and bacterial rRNAs. Maintenance of helix length of 10 bp in the coaxially stacked compound helices and length difference of the pseudoknot helices in the 540 regions in eukaryotic 18S and bacterial 16S rRNAs.

Based on the comparative analysis of the two helices in the 540 region of the SSU rRNA – 500–504/541–545 and 511–515/536–540, it was proposed that these two helices will coaxially stack onto one another. The bacteria have two 5 base pair helices flanking the bulge loop while the eukaryotes and archaea have 6 base pairs in the lower helix and 4 base pairs in the helix above the bulge loop [28] to maintain a total length of 10 base pairs. The intervening bulge loop between its two constituent helices forms a short pseudoknot helix with the terminal hairpin loop (**Figure 3**). However, the crystal structures for the *T. thermophila* and *S. cerevisiae* revealed that these two eukaryotes (and probably all eukaryotes) have 5 base pairs in the lower and upper helices. The sixth ‘putative’ base pair (G:C) in the lower helix did not have any covariation in the eukaryotes and the archaea. Interestingly, the upper helix contains two single nucleotide bulges in the *T. thermophila* SSU and one single nucleotide bulge in the *S. cerevisiae* SSU rRNA (**Figure 3**). It is also interesting to note that the pseudoknot helix between positions 505–507/524–526 in the eukaryotic SSU rRNAs contain only two base pairs, not the three base pairs observed in the bacterial SSU rRNA. Nonetheless, the overall 3D folding pattern of the 540 region in the eukaryotic crystal structures are very similar to that in the bacterial crystal structure (data not shown).

## Discussion

From the analysis of the eukaryotic ribosomal crystal structures for *T. thermophila* and *S.cerevisiae*, and the subsequent comparison with the bacterial high-resolution ribosomal crystal structures, we determined that: (1) nearly all of the base pairs in the eukaryotic SSU rRNA comparative structure model are substantiated. This includes, those base pairs that are in the structure conserved in the three primary phylogenetic domains, those structural elements unique to the eukaryotes, and the non-canonical base pairs that occur in several of the irregular helices (e.g. 39–46/395–403 and the second helix in V3). (2) resolved the secondary structure in the part of the V4 region that had been unstructured in the Gutell lab's eukaryotic comparative structure models. This includes both regular and irregular helices containing an abundance of non-canonical base pairs. (3) Identified tertiary structure interactions in the eukaryotic crystal structures that were not predicted with comparative analysis and determined the binding sites for a few of the eukaryotic ribosomal proteins that interact with regions of the SSU rRNA that are unique to the eukaryotes. Nearly all of the tertiary structure interactions are also present in the bacterial crystal structure. The few exceptions are for those interactions that are interacting between two eukaryotic specific regions. (4) Identified three structural elements, one in V2, one in V4, and the third in V6, that are analogous to bacterial helices. The first two of these helices do not have any obvious simarility between the eukaryotic and bacterial versions. All three were determined to be analogous based on similar tertiary structure interactions and ribosomal protein binding sites. (5) Determined that the four eukaryotic specific ribosomal proteins bind to regions of the SSU rRNA that are unique to the eukaryotes. (6) Determined that the two helices in the 540 region of the SSU rRNA - 500–504/541–545 and 511–515/536–540 each have five base pairs in the eukaryotes, in contrast with the comparative structure models that had six base pairs in the first helix and four base pair in the second. While the bacteria has three base pairs in the 505–507/524–526 pseudoknot helix, the two eukaryotic crystal structures contain only two base pairs.

## Materials and Methods

The RasMol program [29], [30] was used for a detailed visual mapping of the base pairs,long-range tertiary contacts, and RNA-protein interactions in the two eukaryotic (PDB IDs 2XZM and 3U5B/3U5C) and one bacterial (PDB ID 1FJG) SSU rRNA crystal structures [9], [12]. All the figures and supplementary information in the text are also available at http://www.rna.ccbb.utexas.edu/SIM/4A/Hypervariable_SSU_rRNA/ at the CRW Site.

## Supporting Information

Figure S1 **Long-range RNA tertiary contacts in the bacterial** ***Thermus thermophilus*** **SSU rRNA.** This figure, with the long-range tertiary interactions in **Figure 1** shown in thick lines, was generated with RNA2DMap (http://www.rna.icmb.utexas.edu/SAE/2A/RNA2DMap/index.php), a visualization tool based on the RNA secondary structure diagram for displaying both RNA crystal structure information and comparative data.
(EPS)

## References

[1] Noller HF, Chaires JB (1972). Functional modification of 16S ribosomal RNA by kethoxal.. Proc Natl Acad Sci USA.

[2] Hansen JL, Schmeing TM, Moore PB, Steitz TA (2002). Structural insights into peptide bond formation.. Proc Natl Acad Sci USA.

[3] Wimberly BT, Guymon R, McCutcheon JP, White SW, Ramakrishnan V (1999). A detailed view of a ribosomal active site: the structure of the L11-RNA complex.. Cell.

[4] Gutell RR, Weiser B, Woese CR, Noller HF (1985). Comparative anatomy of 16-S-like ribosomal RNA.. Prog Nucleic Acid Res Mol Biol.

[5] Winker S, Woese CR (1991). A definition of the domains Archaea, Bacteria and Eucarya in terms of small subunit ribosomal RNA characteristics.. Syst Appl Microbiol.

[6] Neefs JM, De Wachter R (1990). A proposal for the secondary structure of a variable area of eukaryotic small ribosomal subunit RNA involving the existence of a pseudoknot.. Nucleic Acids Res.

[7] Cannone JJ, Subramanian S, Schnare MN, Collett JR, D'Souza LM (2002). The comparative RNA web (CRW) site: an online database of comparative sequence and structure information for ribosomal, intron, and other RNAs.. BMC Bioinformatics.

[8] Gutell RR, Lee JC, Cannone JJ (2002). The accuracy of ribosomal RNA comparative structure models.. Curr Opin Struct Biol.

[9] Wimberly BT, Brodersen DE, Clemons WM, Morgan-Warren RJ, Carter AP (2000). Structure of the 30S ribosomal subunit.. Nature.

[10] Ban N, Nissen P, Hansen J, Moore PB, Steitz TA (2000). The complete atomic structure of the large ribosomal subunit at 2.4 A resolution.. Science.

[11] Rabl J, Leibundgut M, Ataide SF, Haag A, Ban N (2011). Crystal structure of the eukaryotic 40S ribosomal subunit in complex with initiation factor 1.. Science.

[12] Ben-Shem A, Garreau de Loubresse N, Melnikov S, Jenner L, Yusupova G (2011). The structure of the eukaryotic ribosome at 3.0 A resolution.. Science.

[13] Woese CR, Gutell RR (1989). Evidence for several higher order structural elements in ribosomal RNA.. Proc Natl Acad Sci USA.

[14] Zwieb C, Glotz C, Brimacombe R (1981). Secondary structure comparisons between small subunit ribosomal RNA molecules from six different species.. Nucleic Acids Res.

[15] Nelles L, Van Broeckhoven C, De Wachter R, Vandenberghe A (1984). Location of the hidden break in large subunit ribosomal RNA of Artemia salina.. Naturwissenschaften.

[16] Herzog M, Maroteaux L (1986). Dinoflagellate 17S rRNA sequence inferred from the gene sequence: Evolutionary implications.. Proc Natl Acad Sci USA.

[17] Gonzalez IL, Schmickel RD (1986). The human 18S ribosomal RNA gene: evolution and stability.. Am J Hum Genet.

[18] Ellis RE, Sulston JE, Coulson AR (1986). The rDNA of *C. elegans*: sequence and structure.. Nucleic Acids Res.

[19] Rairkar A, Rubino HM, Lockard RE (1988). Chemical probing of adenine residues within the secondary structure of rabbit 18S ribosomal RNA.. Biochem.

[20] Hendriks L, De Baere R, Van Broeckhoven C, De Wachter R (1988). Primary and secondary structure of the 18 S ribosomal RNA of the insect species Tenebrio molitor.. FEBS Lett.

[21] Johansen T, Johansen S, Haugli FB (1988). Nucleotide sequence of the *Physarum polycephalum* small subunit ribosomal RNA as inferred from the gene sequence: secondary structure and evolutionary implications.. Curr Genet.

[22] Nickrent DL, Sargent ML (1991). An overview of the secondary structure of the V4 region of eukaryotic small-subunit ribosomal RNA.. Nucleic Acids Res.

[23] Nelles L, Fang BL, Volckaert G, Vandenberghe A, De Wachter R (1984). Nucleotide sequence of a crustacean 18S ribosomal RNA gene and secondary structure of eukaryotic small subunit ribosomal RNAs.. Nucleic Acids Res.

[24] Alkemar G, Nygard O (2003). A possible tertiary rRNA interaction between expansion segments ES3 and ES6 in eukaryotic 40S ribosomal subunits.. RNA.

[25] Spahn CM, Beckmann R, Eswar N, Penczek PA, Sali A (2001). Structure of the 80S ribosome from *Saccharomyces cerevisiae*–tRNA-ribosome and subunit-subunit interactions.. Cell.

[26] Alkemar G, Nygard O (2004). Secondary structure of two regions in expansion segments ES3 and ES6 with the potential of forming a tertiary interaction in eukaryotic 40S ribosomal subunits.. RNA.

[27] Lecompte O, Ripp R, Thierry JC, Moras D, Poch O (2002). Comparative analysis of ribosomal proteins in complete genomes: an example of reductive evolution at the domain scale.. Nucleic Acids Res.

[28] Gutell RR, Larsen N, Woese CR (1994). Lessons from an evolving rRNA: 16S and 23S rRNA structures from a comparative perspective.. Microbiol Rev.

[29] Sayle RA, Milner-White EJ (1995). RASMOL: biomolecular graphics for all.. Trends Biochem Sci.

[30] Bernstein HJ (2000). Recent changes to RasMol, recombining the variants.. Trends Biochem Sci.

